# Emodin, Physcion, and Crude Extract of *Rhamnus sphaerosperma* var. *pubescens* Induce Mixed Cell Death, Increase in Oxidative Stress, DNA Damage, and Inhibition of AKT in Cervical and Oral Squamous Carcinoma Cell Lines

**DOI:** 10.1155/2018/2390234

**Published:** 2018-07-03

**Authors:** Thais Fernanda Moreira, Juliana Maria Sorbo, Felipe de Oliveira Souza, Barbara Colatto Fernandes, Fernanda Maria Marins Ocampos, Daniella Maria Soares de Oliveira, Carlos Alberto Arcaro, Renata Pires Assis, Andersson Barison, Obdulio Gomes Miguel, Amanda Martins Baviera, Christiane Pienna Soares, Iguatemy Lourenço Brunetti

**Affiliations:** ^1^Department of Clinical Analysis, School of Pharmaceutical Sciences, São Paulo State University (UNESP), 14800-903 Araraquara, SP, Brazil; ^2^Department of Pharmacy, Federal University of Paraná (UFPR), 80210-170 Curitiba, PR, Brazil; ^3^Department of Chemistry, Federal University of Paraná (UFPR), 80210-170 Curitiba, PR, Brazil

## Abstract

There have been few studies on the pharmacological properties of *Rhamnus sphaerosperma* var. *pubescens*, a native Brazilian species popularly known as “fruto-de-pombo.” The aim of this study was to investigate the scavenging capacity of emodin, physcion, and the ethanolic crude extract of *Rhamnus sphaerosperma* var. *pubescens* against reactive oxygen and nitrogen species, as well as their role and plausible mechanisms in prompting cell death and changes in AKT phosphorylation after cervical (SiHa and C33A) and oral (HSC-3) squamous cell carcinoma treatments. Emodin was shown to be the best scavenger of NO^•^ and O_2_^•−^, while all samples were equally effective in HOCl/OCl^−^ capture. Emodin, physcion, and the ethanolic extract all exhibited cytotoxic effects on SiHa, C33A, HSC-3, and HaCaT (immortalized human keratinocytes, nontumorigenic cell line), involving mixed cell death (apoptosis and necrosis) independent of the caspase activation pathway. Emodin, physcion, and the ethanolic extract increased intracellular oxidative stress and DNA damage. Emodin decreased the activation of AKT in all tumor cells, physcion in HSC-3 and HaCaT cells, and the ethanolic extract in C33A and HaCaT cells, respectively. The induction of cancer cell death by emodin, physcion, and the ethanolic crude extract of *Rhamnus sphaerosperma* var. *pubescens* was related to an increase in intracellular oxidative stress and DNA damage and a decrease in AKT activation. These molecules are therefore emerging as interesting candidates for further study as novel options to treat cervical and oral carcinomas.

## 1. Introduction

Cancer is a major global health concern. High morbidity and mortality rates indicate an increase in the global incidence of cancer, mainly owing to aging populations. Cervical cancer is the fourth most common cancer diagnosed in women worldwide; it is associated with human papillomavirus (HPV) infection. Despite vaccination efforts against HPV infections, since vaccines may provide cross-protection against some HPV strains known to cause cervical cancer, a considerable number of female deaths is still attributed to cervical cancer [1].

HPV has often been associated with oncogenesis, since it causes genetic and metabolic changes that favor tumor development. Its targets are p53, retinoblastoma protein (pRb), and the PI3K/AKT pathway. Thus, in addition to cervical cancer, HPV is associated with the induction of other types of cancer, including squamous cell carcinoma of the esophagus and oral cavity (oropharynx, tonsils, and tongue) [1–4].

The PI3K/AKT signaling pathway is important in regulating normal cell processes, such as proliferation, motility, survival, and cell death. Deregulation of this pathway contributes to tumorigenesis in many cancers, including the squamous cell carcinomas. Alterations in AKT, PIK3CA (which encodes for the p110*α* catalytic subunit of PI3K), and PTEN have been described in squamous cell carcinomas of oral origin (HSC-2, HSC-3, and HSC-4), as well as in cell carcinomas of cervical origin (HeLa, CaSki, SiHa, and C33A) [5–8].

Hyperactivation of the PI3K/AKT pathway in tumor cells leads to a continuous flow of substrates through the glycolytic pathway, contributing with the Warburg effect, (increased glucose uptake and lactate production, even in the presence of oxygen and mitochondrial metabolism) which is highly dependent on complete AKT activation. Complete activation of AKT requires PI3K activity and phosphorylation of both the Thr-308 residue by PDK-1 and the Ser-473 residue by mTORC2. In contrast, PTEN acts as a tumor suppressor and plays an essential role in inhibiting PI3K/AKT signaling [9–12]. AKT regulates the cell cycle and proliferation directly by acting on CDKI (kinase-dependent cyclin inhibitors), such as p21 and p27, and indirectly by modulating the levels of cyclin D1 and p53. AKT also promotes the phosphorylation and inactivation of transcriptional factors FOXO (Forkhead box O); FOXO factors act directly on the cell cycle, DNA repair, and apoptosis, and their inactivation promotes a decrease in the expression of negative regulators of the cell cycle, such as the proteins related to retinoblastoma, p130, CDKI, and p27 [13].

In the metabolic state of neoplastic cells, RONS, such as superoxide anion (O_2_^•**−**^), hydrogen peroxide (H_2_O_2_), and nitric oxide (^•^NO), occur abundantly. The effects of RONS can vary depending on their concentrations in the cells. Intracellular nitric oxide (^•^NO) causes inactivation of PTEN through S-nitrosylation and consequently ubiquitin-mediated proteasomal degradation. Changes in the PTEN status are associated with the redox status and are important for cell survival and proliferation [14]. In these cells, RONS levels are controlled via antioxidant defenses. An increase in NADPH production by glutamine metabolism and the pentose phosphate pathway facilitate glutathione (GSH) regeneration as well as the expression of enzymes that act on RONS metabolism, such as catalase, SOD, NOX-1, and DUOX-POD [15–17].

Inhibition of the PI3K/AKT pathway culminates in the loss of regulation of mechanisms involved in tumor cell proliferation and survival, thus emerging as an important therapeutic target for tumor suppression. Compounds able to unbalance the redox state and to promote alterations in the PI3K/AKT pathway may be useful to induce cell death in tumor cells.

Anti-inflammatory, antioxidant, antihypertensive, antimutagenic, and apoptosis-inducing properties have been described for species of the genus *Rhamnus* [18–20]. In our previous study, chrysophanol and physcion (anthraquinones) and stigmasterol and sitosterol (phytosterols) were isolated from the ethanolic extract of *R. sphaerosperma* var. *pubescens* stems; the extract and its fractions exhibited antioxidant properties and the ability to inhibit liporoxidation [21].

Anthraquinones have a chemical structure similar to that of anthracyclines, a class of chemotherapeutic agents such as doxorubicin, which demonstrate effectiveness in several tumor types [22]. These chemical properties of anthraquinones, combined with molecular modeling and other tools, together contribute to the improvement of the therapeutic arsenal against squamous cell carcinoma. From this perspective, the present study identified different responses of the anthraquinones emodin and physcion and crude extracts from *Rhamnus sphaerosperma* var. *pubescens* related to the ability to capture reactive oxygen and nitrogen species. Furthermore, the mechanisms of cell death induction and the changes in AKT phosphorylation levels were analyzed after treatments with emodin, physcion, or ethanolic crude extract of *Rhamnus sphaerosperma* var. *pubescens*, in the following cancer cell lines: SiHa, C33A (cervical squamous cell carcinomas), and HSC-3 (oral squamous cell carcinomas).

## 2. Materials and Methods

### 2.1. Equipment and Chemicals

NMR spectra were recorded on a Bruker Avance III 600® (Bruker, Massachusetts, USA). The capture assays were performed in a microplate spectrophotometer (*PowerWave* XS2, BIOTEK®, Vermont, USA). Fetal bovine serum was purchased from Cultilab®, São Paulo, Brazil. Fluorescein diacetate, propidium iodide, and Hoechst 33342 were purchased from Invitrogen®, Ontario, Canada. Annexin-V conjugated with FITC was purchased from Life Technologies®, Ontario, Canada. Sodium pyrophosphate, sodium fluoride, and sodium nitroprusside were purchased from Merck®, Darmstadt, Germany. The TBARS assay was performed in a microplate spectrofluorimeter (*Synergy 5*, BIOTEK, Vermont, USA). The comet assay was performed in fluorescence microscope (ECLIPSE 50i; Nikon Instruments Inc., Nikon do Brasil LTDA., São Paulo, Brazil) coupled with a photograph camera (DS-Ri1; Nikon Instruments Inc., Nikon do Brasil LTDA., São Paulo, Brazil). Western blot analysis was performed in a Bio-Rad miniature gel apparatus and miniature transfer apparatus (Mini-Protean, Bio-Rad Laboratories, California, EUA), and a C-Digit® Blot Scanner (LI-COR, Nebraska, USA) was used to capture chemiluminescent signals in membranes.

Ultrapure water was used in all assays was obtained in a Milli-Q® Millipore System, USA.

Deuterated solvent chloroform (CDCl_3_) was purchased from Cambridge Isotope Laboratories (Massachusetts, USA). Dimethyl sulfoxide (DMSO, 99.9%, *v*/*v*), trolox, gallic acid, quercetin, curcumin, ascorbic acid, catalase, doxorubicin, Dulbecco's modified Eagle medium, Ham F10 and F12 nutrient mixtures, hydrocortisone penicillin and streptomycin solution, sodium bicarbonate, kanamycin, fluorigenic substrate for caspase-3 (Ac-Asp-Met-Gln-Asp-AMC), fluorescein isothiocyanate, dithiothreitol, Tris, sodium dodecyl sulfate, *β*-mercaptoethanol, aprotinin, PMSF, leupeptin, sodium orthovanadate, NaOCl, 3,3′,5,5′-tetramethylbenzidine, potassium iodide, NADH, phenazinemetasulfate, nitroblue tetrazolium, sulfanilic acid, N-(1-naphthalenediamine), cytochalasin B, and trypsin were purchased from Sigma-Aldrich® (Sigma-Aldrich Brasil Ltda., São Paulo, Brazil). Fetal bovine serum (Cultilab®, São Paulo, Brazil), fluorescein diacetate, propidium iodide and Hoechst 33342 (Invitrogen®, Ontario, Canada), and annexin-V conjugated with FITC (Life Technologies*®*, Ontario, Canada). Sodium pyrophosphate, sodium fluoride, and sodium nitroprusside (Merck®, Darmstadt, Germany).

Antibodies anti-AKT, anti-p-[Ser-473]-AKT, anticaspase-3, anti-BAX, anti-BCL-2, and anti-rabbit IgG conjugated with horseradish peroxidase were purchased from Cell Signaling Technology® (Massachusetts, USA), and anti-GAPDH was purchased from CUSABIO Technology LLC® (Texas, USA).

### 2.2. Plant Material Isolation and Identification

The plant species were collected at the Federal University of Paraná, Curitiba, Paraná, Brazil (25°26′52^″^S49°14′21^″^W), during the morning hours of May 2011 and July 2013. Botanical identification was carried out at the Municipal Botanical Museum of Curitiba (Paraná, Brazil).

Dry and powdered stems of *Rhamnus sphaerosperma* var. *pubescens* were subjected to extraction with absolute ethanol, and the ethanolic crude extract (EERs) was separated into a liquid–liquid partition with hexane (hexane fraction—HFRs), chloroform (chloroform fraction—CFRs), and ethyl acetate (ethyl acetate fraction—EAFRs). Both methods were performed in Soxhlet equipment at 60°C. The hexane fraction was subjected to column chromatography using silica gel 60 and a mobile phase composed of hexane and ethyl acetate, and ethyl acetate and methanol, with gradients of increasing polarities (starting from 100% *v*/*v* hexane to 100% *v*/*v* ethyl acetate and from 100% *v*/*v* ethyl acetate to 80% *v*/*v* methanol).

### 2.3. RONS Capture Assays

#### 2.3.1. O_2_^•−^ Capture Assay

This method is based on the *in vitro* formation of O_2_^**•−**^ via nonenzymatic reactions between reduced nicotinamide adenine dinucleotide (NADH), phenazinemetasulfate (PMS), and oxygen dissolved in the medium. The reactive species formed promotes the reduction of nitroblue tetrazolium (NBT), generating a formazane salt, which is quantified by spectrophotometry (560 nm). The assay was performed in sodium pyrophosphate buffer (25 mM, pH 8.3), with PMS (0.372 mM), NBT (0.6 mM), NADH (1.56 mM), and various concentrations of the samples, with a final reaction volume of 300 *μ*L. After incubation in the dark at room temperature for 7 min, the absorbance was measured at 560 nm [23].

#### 2.3.2. HOCl/OCl^−^ Capture Assay

HOCl/OCl^−^ has the ability to oxidize 3,3′,5,5′-tetramethylbenzidine (TMB), generating a blue staining compound detected at 652 nm. Initially, the OCl^−^ concentration was determined by its molar extinction coefficient (*ε* = 350 M^−1^ · cm^−1^ at 292 nm) [24]. The TMB solution (2.8 mM) was prepared from its dissolution in dimethylformamide (50%, *v*/*v*), 0.8 M acetic acid (49%, *v*/*v*), and 0.01 M potassium iodide (1%, *v*/*v*). The reaction began with incubation of the samples at different concentrations, diluted in sodium phosphate buffer (50 mM, pH 7.4) with HOCl/OCl^−^ (30 *μ*M), and revealed with TMB (2.8 mM) at a final reaction volume of 300 *μ*L [25–27].

#### 2.3.3. ^•^NO Capture Assay

Nitric oxide, generated from the dissolution of sodium nitroprusside in an aqueous solution with pH 7.4, reacts with oxygen in the medium, generating nitrate and nitrite. The concentration of nitrite is determined spectrophotometrically, by subjecting it to reaction with Griess reagent: 1.0% (*w*/*v*) sulfanilic acid, 0.1% (*w*/*v*) N-(1-naphthalenediamine), and 2.5% (*v*/*v*) phosphoric acid, at 540 nm. The samples were incubated at different concentrations with sodium nitroprusside (4 mM) in a sodium phosphate buffer (20 mM, pH 7.4) for 150 min at room temperature. Then Griess reagent was added, and at a final reaction volume of 300 *μ*L, the absorbance was measured at 540 nm [28].

### 2.4. Assays for Cell Death

#### 2.4.1. Cell Culture

Cell lines SiHa (ATCC® HTB35™), C33A (ATCC HTB31™), and HaCaT (CLS 300493) were grown in DMEM (Dulbecco's modified Eagle medium) with Ham F10 (F10 Ham nutrient mixture). The HSC-3 cell line (JCRB: JCR 0623) was grown in DMEM with Ham F12 (F12 Ham nutrient mixture), hydrocortisone (0.4 *μ*g/L), and ascorbic acid (50 mg/L). All media were supplemented with fetal bovine serum (10% *v*/*v*), penicillin (100 U/mL), streptomycin (100 *μ*g/mL), amphotericin B (0.25 mg/L), kanamycin (0.1 g/L), and HEPES (5.96 g/L). Cultures were grown in a sterile atmosphere at 37°C under 5% CO_2_.

Emodin, physcion, ethanolic extract, and hexane fraction from *Rhamnus sphaerosperma* var. *pubescens* stems were used to study cell death mechanisms. The working concentrations were established by a sulforhodamine B assay (data not shown). Chrysophanol did not induce cytotoxicity for all cell lines studied.

#### 2.4.2. Cytomorphological Viability Assay with Hoechst 33342, Propidium Iodide, and Fluorescein Diacetate

The cells were plated (5 × 10^3^ cells/well) in 96-well plates for 24 h, and the treatments were performed for 12 h or 24 h. After the treatment periods, the plate was centrifuged at 1258*g*, and each well was washed with phosphate buffered saline (PBS) and centrifuged again under the same conditions. The distinction between viable cells and dead cells in apoptosis and necrosis was performed by adding a solution (100 *μ*L/well) containing fluorescein diacetate (3.5 *μ*g/mL), propidium iodide (2.5 *μ*g/mL), and Hoechst 33342 (1.5 *μ*g/mL) in PBS. After incubation for 10 min at room temperature in the dark, the images were acquired and analyzed using an imaging cytometer IN Cell Analyzer 2000 [29].

#### 2.4.3. Annexin-V Cell Death Assay

The cells were plated (5 × 10^3^ cells/well) in 96-well plates for 24 h, and the treatments were performed for 12 h or 24 h. After the treatment period, the plate was centrifuged at 1258*g*, and each well was washed with PBS and centrifuged again under the same conditions. After washing, 100 *μ*L/well of annexin-V binding buffer (10 mM HEPES, 140 mM NaCl, and 2.5 M CaCl_2_) containing annexin-V/FITC (1.3 *μ*g/mL) and Hoechst 33342 (1.6 *μ*g/mL) was added, followed by incubation for 15 minutes at 4°C in darkness. After incubation, 20 *μ*L/well of propidium iodide (1 *μ*g/mL, diluted in the buffer described above) was added. The images were acquired and analyzed using an imaging cytometer IN Cell Analyzer 2000 [30].

#### 2.4.4. Caspase-3 Activity Assay

The cells were plated (1 × 10^4^ cells/well) in 96-well plates for 24 h, and the treatments were performed for 6 h. After the treatment period, the plate was centrifuged at 1258 ×g, and each well was washed with PBS and centrifuged again under the same conditions. After washing, the cells were permeabilized with a 0.5% (*w*/*v*) saponin solution (dissolved in culture medium containing 5 mM DTT and 2 mM EDTA) for 10 min. After the permeabilization step, a solution with caspase-3-specific fluorogenic substrate (20 *μ*M) and propidium iodide (2.5 *μ*g/mL—for nuclear staining) was added (100 *μ*L/well). The images were immediately acquired and analyzed using an imaging cytometer IN Cell Analyzer 2000 [31].

#### 2.4.5. Western Blot Analysis

The cells were plated (8 × 10^5^ cells/well) in 6-well plates for 24 h, and the treatments were performed for 24 h. After the treatment period, the cells were washed with PBS and lysed with lysis solution containing Tris (125 mM, pH 6.8), sodium dodecyl sulfate (10%, *w*/*v*), *β*-mercaptoethanol (1%, *v*/*v*), protease inhibitors (5 *μ*g/mL aprotinin, 1 mM PMSF, and 2.34 mM leupeptin), and phosphatase inhibitors (10 mM sodium pyrophosphate, 100 mM sodium fluoride, and 10 mM sodium orthovanadate). After lysis, the cell lysates were heated to 95°C for 8 min, and the samples were stored at −80°C until use; an aliquot was separated for quantification of total proteins by the Lowry method [32].

Proteins from cell lysates were separated by 10% (*w*/*v*) SDS polyacrylamide gel electrophoresis (SDS-PAGE) [33]. Proteins were transferred to nitrocellulose membranes (Amersham™ Protran™—GE Health Care) [34] and blocked for 1 hour at room temperature with 10% (*w*/*v*) nonfat dried milk in Tris-buffered saline with Tween 20 (10 mM Tris, 125 mM NaCl, and 0.1% *v*/*v* Tween 20). The membranes were then incubated overnight at 4°C with specific primary antibodies: anti-AKT (1 : 500), anti-p-[Ser-473]-AKT (1 : 500), anticaspase-3 (1 : 1000), anti-BAX (1 : 1000), anti-BCL-2 (1 : 1000), and anti-GAPDH (1 : 1000). After washing with Tris-buffered saline and Tween 20, the membranes were incubated with secondary antibodies (HRP-conjugated anti-rabbit IgG, 1 : 1000) for one hour at room temperature. After washing, the membranes were revealed using a chemiluminescent substrate (5 mM luminol, 8 mM hydrogen peroxide, 4 mM p-iodophenylboronic acid, and 0.1 M Tris–HCl; pH 8.8) [35]. Chemiluminescent bands were captured with a C-Digit Blot Scanner (LI-COR, Lincoln, NE), and the band intensities were analyzed using LI-COR Image Studio 4.0.

### 2.5. Oxidative Damage Analysis

#### 2.5.1. TBARS Assay

The cells were plated (2 × 10^5^ cells/well) in 24-well plates for 24 h, and the treatments were performed for 24 h. After the treatment, the culture medium of each well (200 *μ*L) was used to quantify TBARS via reaction with 800 *μ*L of an aqueous mixture of thiobarbituric acid (0.8%, *w*/*v*) diluted with acetic acid (20%, *v*/*v*) and sodium dodecyl sulfate (8.1%, *w*/*v*). The tubes were incubated at 95°C for 60 min, and the fluorescence intensity was measured with excitation and emission wavelengths of 510 and 553 nm, respectively, using 1,1,3,3-tetramethoxypropane as a standard. The results were expressed as *μ*M of TBARS [36].

### 2.6. DNA Damage Analysis

#### 2.6.1. Micronuclei Assay

The cells were plated (4 × 10^3^ cells/well) in 96-well plates for 24 h, and the treatments were performed for 24 h. After treatment, the wells were washed with PBS and incubated with cytochalasin B (6 *μ*g/mL) for 36 h at 37°C to block cytokinesis, allowing observation of multinucleated cells. Thereafter, the cells were fixed with absolute ethanol for 30 minutes and stained with fluorescein isothiocyanate (FITC) (1 *μ*g/mL) for 1 h for cytoplasmic staining and with Hoechst 33342 (10 *μ*g/mL) for 30 min for DNA staining. The images were acquired and analyzed using the imaging cytometer IN Cell Analyzer 2000 [37].

#### 2.6.2. Comet Assay

The cells were plated (1 × 10^5^ cells/well) in 24-well plates for 24 h, and the treatments were performed for 6 h. The comet assay was performed as described by Singh et al. [38] and Tice et al. [39]. For each test (samples and respective concentrations), 50 cells were analyzed using TriTek Comet Score TM version 1.5 software. The percentage of DNA in the tail was used to analyze the amount of fragmented DNA [40].

### 2.7. Statistical Analysis

For cell death and caspase activity assays, one-way analysis of variance (ANOVA) followed by the Tukey's post-test was applied. For the micronuclei, TBARS, and Western blot assays, ANOVA followed by the Newman–Keuls post-test was applied. For the comet assay, a Kruskal-Wallis test with a Dunn's post-test was applied. GraphPad Prism 6.0 software was used in these analyses.

## 3. Results and Discussion

### 3.1. Chrysophanol, Emodin, and Physcion Were Isolated from *R. sphaerosperma* var. *pubescens*

The crude ethanolic extract of *R. sphaerosperma* and its fractions underwent hydrogen magnetic resonance analysis (^1^H NMR). The ^1^H NMR spectra showed peaks corresponding to anthraquinone nuclei, except in the ethyl acetate fraction. Characterization of the anthraquinone nucleus by ^1^H NMR is defined by chemical shifts in the region of 10–12 ppm of the spectrum, corresponding to hydroxyl groups chelated by hydrogen bonds, and chemical shifts between 6 and 7 ppm, characteristic of ortho- and metacoupling of hydrogen [41]. After column chromatography of the hexane fraction, three crystalline samples were isolated and identified with ^1^H NMR as chrysophanol (1,8-dihydroxy-3-methylanthraquinone), physcion (1,8-dihydroxy-6-methoxy-3-methylanthraquinone), and emodin (1,6,8-trihydroxy-3-methylanthraquinone). The chemical shift and coupling constant values of the ^1^H NMR spectra for the anthraquinones are shown in Table 1.

To the best of our knowledge, the present study is the first to isolate these compounds in *R. sphaerosperma* var. *pubescens*. However, they are very common in other species of the genus *Rhamnus* (e.g., *Rhamnus frangula*), in addition to several other plant and animal species, as well as microorganisms (fungi, bacteria, and lichens) and insects [41].

### 3.2. Samples from *R. sphaerosperma* var. *pubescens* Exhibited Capture Capacity for O_2_^•−^, HOCl/OCl^−^_,_ and ^•^NO

The effective concentration that promotes 50% inhibition (EC_50_) in the capture of the reactive species was calculated by linear regression. These values for samples and standards were statistically compared for each test, with lower EC_50_ values indicating greater effectiveness. These results are presented in Table 2. Curcumin (*β*-diketone) and quercetin (flavonoid) showed higher efficiency for all reactive species studied. Gallic acid (phenolic acid) showed lower efficiency against ^•^NO, and Trolox (tocopherol's water-soluble analog) showed higher EC_50_ (lower efficiency) in the capture of HOCl/OCl^−^ and O_2_^•−^, and there is no activity on ^•^NO (Table 2).

Among all compounds used as controls in assays to assess RONS capture capacity, curcumin is the most well known for its cytotoxic capacity. It has been shown to be effective in the induction of cell death in various tumor cell lines [42]; therefore, it was also used as a control in the cell death assays.

RONS capture capacity has been reported for several biomolecules, since RONS are determinants of metabolic and proliferative controls, most notably in tumor cells [43].

Oncogenic transformation is functionally associated with the expression of cell membrane enzymes, such as NOX1, which generates O_2_^•**−**^ that undergoes spontaneous or catalyzed (by SOD) dismutation to H_2_O_2_, which is decomposed into H_2_O and O_2_ by the action of catalase; the conversion of H_2_O_2_ into HOCl occurs by the action of free peroxidase released by DUOX-POD, in the presence of chloride. This module of NOX1/SOD/catalase/DUOX-POD enzymes has been found in bona fide tumor cells derived from different tissues. Selective signaling for apoptosis in transformed cells has been reported in dominant pathways, such as those signaled by HOCl/OCl^−^ and ^•^NO/ONOO^−^, and in less important pathways, such as the nitryl chloride and Fenton/Haber-Weiss reactions. Based on (i) peroxidase action, (ii) the dominant processes that generates peroxynitrite in a reaction between ^•^NO and O_2_^•**−**^ and the decomposition of peroxynitrous acid into ^•^NO_2_ and ^•^OH, and (iii) the reaction between HOCl and O_2_^•**−**^ to produce ^•^OH, Cl^**−**^, and O_2_, the ^•^OH causes lipoperoxidation and triggers the onset of the mitochondrial pathway of apoptosis [44, 45].

The ethyl acetate fraction of *Rhamnus sphaerosperma* var. *pubescens* (EAFRs) was more efficient for HOCl/OCl^−^ and ^•^NO capture, yet both the crude extract (EERs) and EAFRs exhibit similar behavior for the capture of O_2_^•**−**^. These samples are the most effective of those isolated from *R. sphaerosperma* var. *pubescens*. For the ethanolic crude extract and fractions, the presence of anthraquinones in their composition did not influence their capture capacity. EAFRs have no anthraquinones and presented better efficacy when compared to the other fractions that contain anthraquinone compounds, showing equivalency to the crude extract. Thus, it is possible that the capture capacity of the ethanolic crude extract and its fractions is due to the combined effects and/or synergism of compounds not yet identified.

Emodin showed better O_2_^•−^ and ^•^NO capture capacity, while other anthraquinones were not active for this species. In the HOCl/OCl^−^ capture assay, there were no statistically significant differences among anthraquinones (Table 2). Few structural differences are observed in these compounds; the presence of an electron-donor group (methoxyl) in the structure of physcion worsens the capture efficiency of the studied reactive species in comparison with an electron-acceptor group (hydroxyl) in the meta position of the emodin structure. The quinone nucleus of the anthraquinones possesses recognized redox capacity, with intermediate formation of the semiquinone radical and subsequent stabilization in hydroquinone [46–48].

Based on these data, we would expect that the treatment of tumor cells with emodin can result in the capture of these reactive species (O_2_^•−^ and/or ^•^NO) in the cell environment, causing changes in RONS signaling, which might alter the balance and status of cell survival and proliferation. Additionally, with the formation of semiquinone, toxicity may also occur. The same cannot be attributed to physcion, which was efficient only in capturing HOCl/OCl^−^; nevertheless, it also shows relevant cytotoxicity in cell death assays. Conversely, chrysophanol, which is also efficient in capturing HOCl/OCl^−^, was not able to exert cytotoxicity, suggesting that the semiquinones produced from the interaction of these anthraquinones with cellular RONS have different cytotoxic capacities. However, further experiments are necessary to explore these hypotheses.

### 3.3. Emodin, Physcion, EERs, and HFRs Induced Mixed Cell Death Independent of Caspase-3 Activation

The cytotoxic profile of *R. sphaerosperma* var. *pubescens* was analyzed against different normal cell lines (HaCaT) or tumor cell lines (SiHa, C33A, and HSC-3) by the sulforhodamine B assay (data not shown). Chrysophanol and the EAFRs did not exert toxic effects on the evaluated cell lines, while emodin, physcion, ethanolic crude extract (EERs), and its hexane fraction (HFRs) showed higher cytotoxic effects; therefore, the latter were selected for additional cell death assays.

Emodin was studied at concentrations from 46.3 to 185.0 *μ*M (12.5 to 50.0 *μ*g/mL), physcion was studied at concentrations from 43.8 to 175.0 *μ*M (12.5 to 50.0 *μ*g/mL), and ethanolic crude extract (EERs) and its hexane fraction (HFRs) were evaluated at concentrations from 25.0 to 100.0 *μ*g/mL. For the SiHa and C33A cell lines, the time of treatment was 24 h, and for the HSC-3 and HaCaT cell lines, it was 12 h. As positive controls for all cell lines, curcumin induced predominantly apoptosis and doxorubicin induced necrosis.

**Figure 1:**
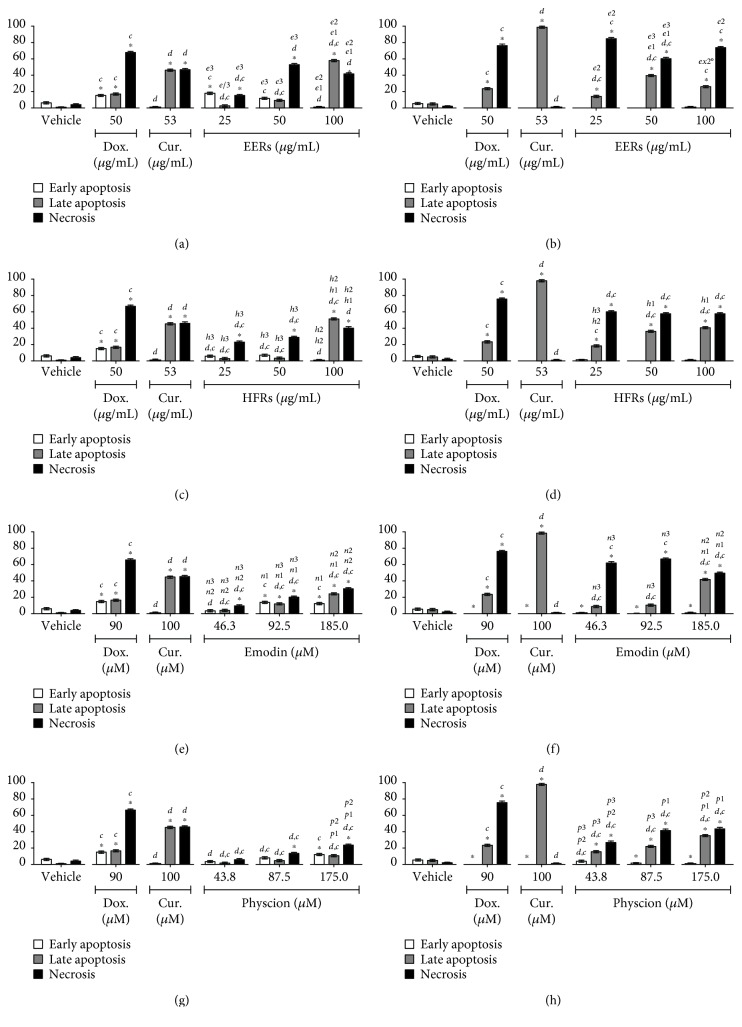
Cell death analysis in SiHa cells. Cytomorphological viability assay for (a) EERs, (c) HFRs, (e) emodin, and (g) physcion. Annexin-V assay for (b) EERs, (d) HFRs, (f) emodin, and (h) physcion. Comparison with significant statistical difference between treatments: vehicle (^∗^); doxorubicin (d); curcumin (c); EERs: 25 *μ*g/mL (e1); EERs: 50 *μ*g/mL (e2); EERs: 100 *μ*g/mL (e3); HFRs: 25 *μ*g/mL (h1); HFRs: 50 *μ*g/mL (h2); HFRs: 100 *μ*g/mL (h3); emodin: 46.3 *μ*M (n1); emodin: 92.8 *μ*M (n2); emodin: 185.0 *μ*M (n3); physcion: 43.8 *μ*M (p1); physcion: 87.5 *μ*M (p2); physcion: 175.0 *μ*M (p3); and cell status: early apoptosis, late apoptosis, and necrosis. Results are expressed as mean of three independent experiments ± standard error (*M* ± SE), analyzed by one-way ANOVA with Tukey's pos-ttest, *p* ≤ 0.05.

**Figure 2:**
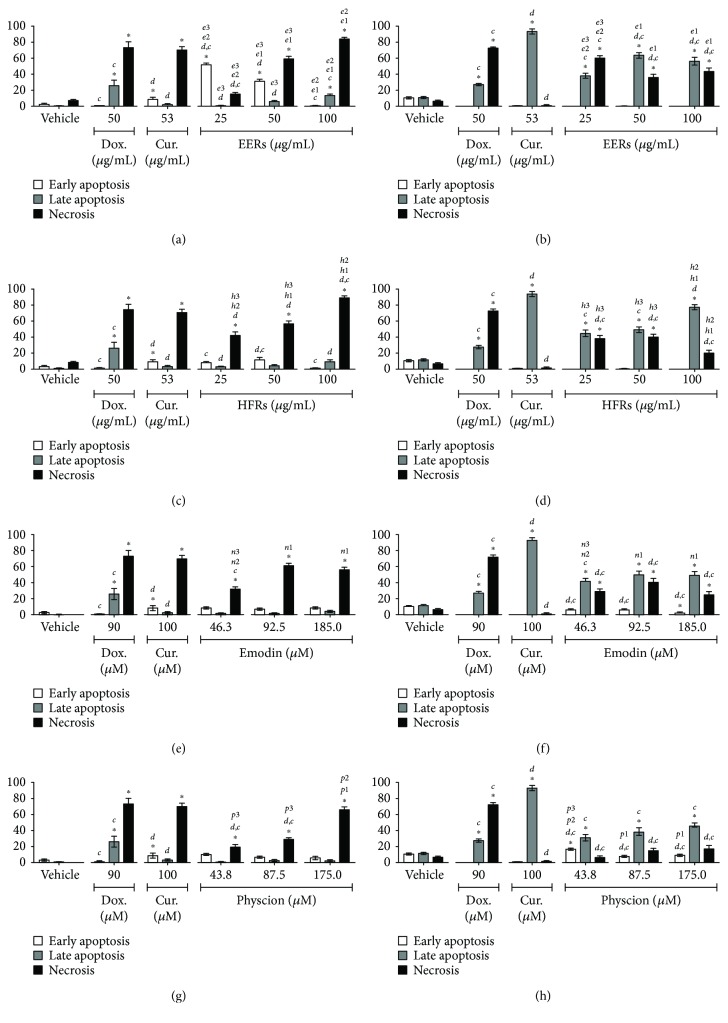
Cell death analysis in C33A cells. Cytomorphological viability assay for (a) EERs, (c) HFRs, (e) emodin, and (g) physcion. Annexin-V assay for (b) EERs, (d) HFRs, (f) emodin, and (h) physcion. Comparison with significant statistical difference between treatments: vehicle (^∗^); doxorubicin (d); curcumin (c); EERs: 25 *μ*g/mL (e1); EERs: 50 *μ*g/mL (e2); EERs: 100 *μ*g/mL (e3); HFRs: 25 *μ*g/mL (h1); HFRs: 50 *μ*g/mL (h2); HFRs: 100 *μ*g/mL (h3); emodin: 46.3 *μ*M (n1); emodin: 92.8 *μ*M (n2); emodin: 185.0 *μ*M (n3); physcion: 43.8 *μ*M (p1); physcion: 87.5 *μ*M (p2); physcion: 175.0 *μ*M (p3); and cell status: early apoptosis, late apoptosis, and necrosis. Results are expressed as mean of three independent experiments ± standard error (*M* ± SE), analyzed by one-way ANOVA with Tukey's post-test, *p* ≤ 0.05.

All tested samples showed a mixed cell death profile for the SiHa cell line, with the presence of cells that were classified as morphologically and biochemically in apoptosis (early or late) and necrosis; emodin was more effective than physcion (Figure 1). For C33A cells, the cytomorphological assay revealed cell death predominantly with necrosis features, while in the annexin-V assay, the presence of apoptosis was observed. All samples were more efficient in the C33A cell line than in SiHa, except for physcion (Figure 2).

**Figure 3:**
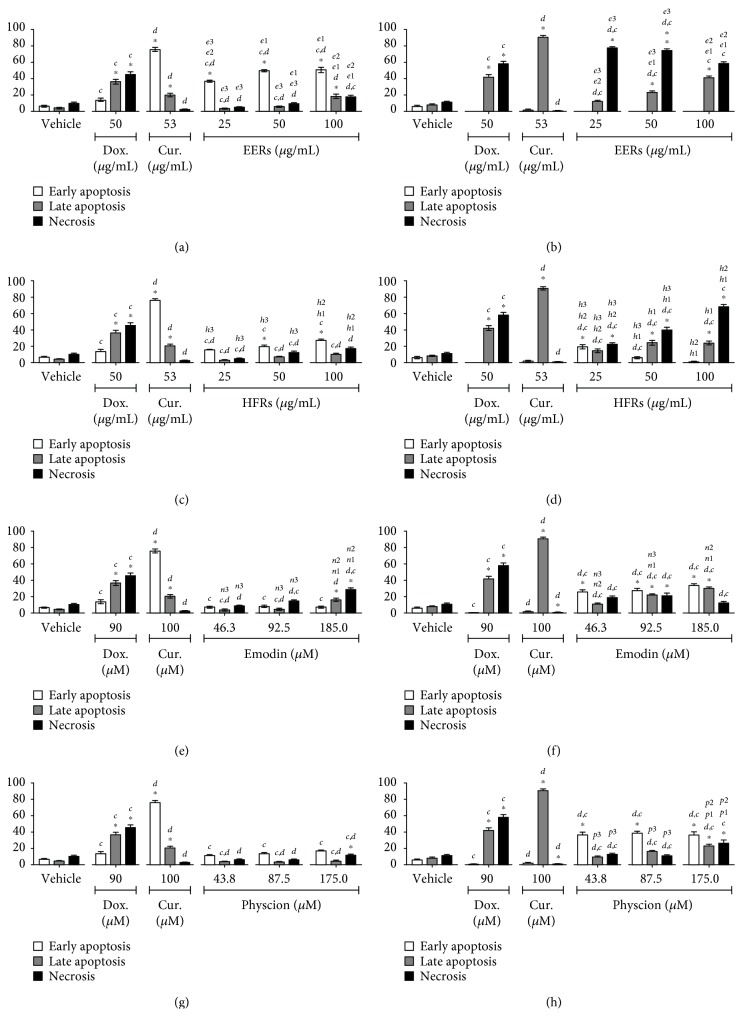
Cell death analysis in HSC-3 cells. Cytomorphological viability assay for (a) EERs, (c) HFRs, (e) emodin, and (g) physcion. Annexin-V assay for (b) EERs, (d) HFRs, (f) emodin, and (h) physcion. Comparison with significant statistical differences between treatments: vehicle (^∗^); doxorubicin (d); curcumin (c); EERs: 25 *μ*g/mL (e1); EERs: 50 *μ*g/mL (e2); EERs: 100 *μ*g/mL (e3); HFRs: 25 *μ*g/mL (h1); HFRs: 50 *μ*g/mL (h2); HFRs: 100 *μ*g/mL (h3); emodin: 46.3 *μ*M (n1); emodin: 92.8 *μ*M (n2); emodin: 185.0 *μ*M (n3); physcion: 43.8 *μ*M (p1); physcion: 87.5 *μ*M (p2); physcion: 175.0 *μ*M (p3); and cell status: early apoptosis, late apoptosis, and necrosis. Results are expressed as mean of three independent experiments ± standard error (*M* ± SE), analyzed by one-way ANOVA with Tukey's posttest, *p* ≤ 0.05.

**Figure 4:**
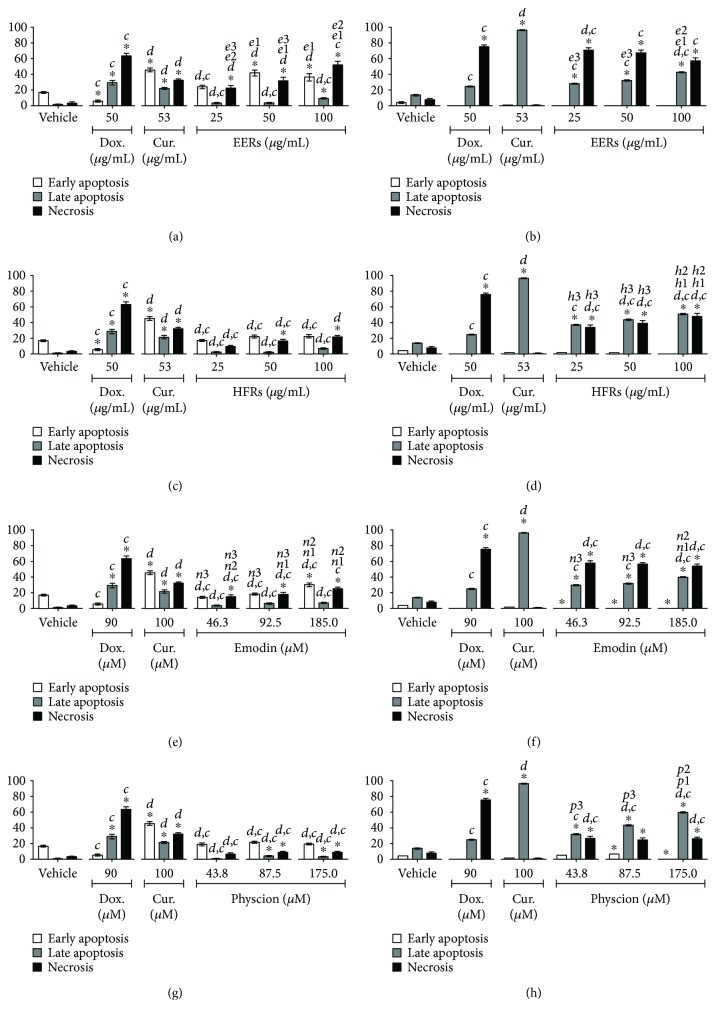
Cell death analysis in HaCaT cells. Cytomorphological viability assay for (a) EERs, (c) HFRs, (e) emodin, and (g) physcion. Annexin-V assay for (b) EERs, (d) HFRs, (f) emodin, and (h) physcion. Comparison with significant statistical differences between treatments: vehicle (^∗^); doxorubicin (d); curcumin (c); EERs: 25 *μ*g/mL (e1); EERs: 50 *μ*g/mL (e2); EERs: 100 *μ*g/mL (e3); HFRs: 25 *μ*g/mL (h1); HFRs: 50 *μ*g/mL (h2); HFRs: 100 *μ*g/mL (h3); emodin: 46.3 *μ*M (n1); emodin: 92.8 *μ*M (n2); emodin: 185.0 *μ*M (n3); physcion: 43.8 *μ*M (p1); physcion: 87.5 *μ*M (p2); physcion: 175.0 *μ*M (p3); and cell status: early apoptosis, late apoptosis, and necrosis. Results are expressed as mean of three independent experiments ± standard error (*M* ± SE), analyzed by one-way ANOVA with Tukey's post-test, *p* ≤ 0.05.

In HSC-3 cells, EERs was more effective than HFRs, with mixed cell death. The anthraquinones were less efficient than EERs and HFRs, while emodin and physcion exhibited no differences in this cell line with regard to the induction of mixed cell death (Figure 3). Cell death induced in HaCaT (normal cell line) was mixed, with both apoptosis and necrosis, except for EERs that induced necrosis in all tested concentrations. All samples were more toxic in HaCaT than HSC-3 (tumor cell line), after 12 hours of treatment (Figure 4).

**Figure 5:**
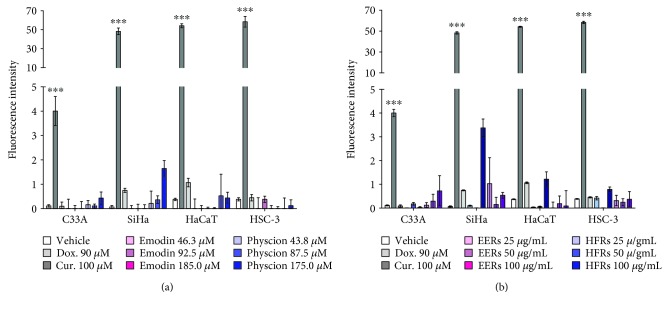
Caspase 3 activity assay. SiHa, C33A, HSC-3, and HaCaT after treatment for 6 h with (a) emodin, physcion and (b) EERs and HFRs. Results are expressed as mean cell fluorescence intensity of three independent experiments ± standard error (*M* ± SE), analyzed by one-way ANOVA with Tukey's post-test (statistically significant difference compared to vehicle control group: ^∗∗∗^*p* ≤ 0.001).

Caspase-3 activity is a hallmark of classical apoptosis. Treatments with anthraquinones, EERs, and HFRs did not promote significant changes in caspase-3 activity (Figure 5); thus, it can be suggested that cell death occurs independently of the caspase-3 pathway and that the morphological and biochemical alterations observed are due to the activation of other biochemical pathways, such as caspase-1 activation, release of interleukins (IL-1*β* and IL-18), and caspase-7 activation, culminating in an unusual process of cell death in which cells may exhibit morphological characteristics of apoptosis and/or necrosis [49]. In the assay of the present study, only the cells treated with curcumin showed activation of caspase-3; treatment with doxorubicin did not change the caspase-3 activity (Figure 5).

To the best of our knowledge, the present study is the first to describe cell death promoted by complex mixtures of the extract and fractions from *Rhamnus sphaerosperma* var. *pubescens*. However, emodin and physcion have previously been recognized for their promotion of cell death. In lung tumor cells A549, H460, and CH27, 10 *μ*M emodin inhibited cell growth after 48 hours of treatment and 50 *μ*M emodin induced apoptosis with DNA fragmentation. In A549 cells, 50 *μ*M emodin also induced activation of caspases-2, -3, and -9 after 12 h of treatment [50]. Caspase-dependent cell death was also observed in Bu 25TK (cervical cell line) and SMMC-7721 and HepG2 (hepatocellular carcinomas) [51, 52].

Some studies have demonstrated the cytotoxic effects of physcion, dependent on the activation of caspases, and cell cycle arrest in HeLa (cervical cancer cells), SW620 (colorectal cancer cells), and MDA-MB-231 (breast cancer cells) [53–55]. Studies using physcion-8-O-*β* glucopyranoside have also shown significant cytotoxic activity in HepG2, A549, and HEM (human melanoma cells) [56–58].

Thus, the findings of the present study revealed that emodin and physcion induced mixed cell death, independent of caspases, in cervical cell lines (SiHa and C33A), after 24 h of treatment, and in an oral cell line (HSC-3) and immortalized keratinocytes (HaCaT) after 12 h of treatment.

### 3.4. Emodin, Physcion, and EERs Increased Intracellular Oxidative Stress

Under normal physiological conditions, cells maintain a balance between oxidant species and antioxidants, referred to as redox homeostasis. At low concentrations, RONS act to regulate proliferation, cell death, and other metabolic processes [59]. An increase in oxidative stress has been associated with several types of cell death, including oxeiptosis, a recently described cell death pathway exhibiting the morphological features of apoptosis that is independent of caspase activation [60].

Studies have associated the toxic effect of emodin and physcion with an increase in intracellular RONS, which thereby promotes deregulation of mitochondrial membrane potential and activation of cell death [50, 52, 53, 56]. Therefore, the levels of thiobarbituric acid reactive species (TBARS) were quantified after the treatment of the cells with emodin, physcion, and EERs.

**Figure 6:**
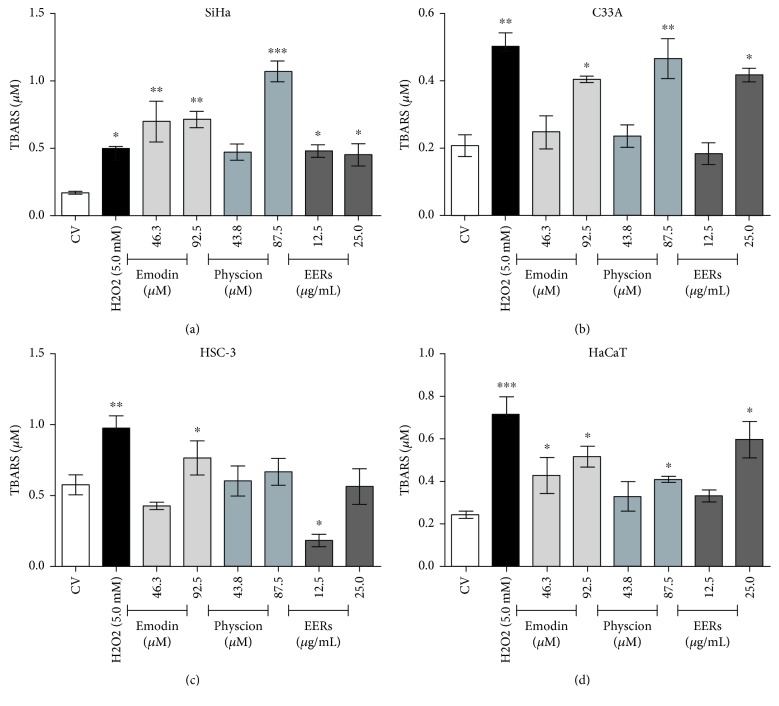
TBARS assay in SiHa, C33A, HSC-3, and HaCaT. 24-hour treatment with emodin, physcion, and EERs. H_2_O_2_ as positive control (5.0 mM). Results are expressed as mean of TBARS concentration (product formed after the reaction of the extracellular medium with thiobarbituric acid in at 95°C for 60 min) of three independent experiments ± standard error (*M* ± SE), analyzed by one-way ANOVA and Newman–Keuls post-test (statistically significant difference compared to the vehicle control group: ^∗^*p* ≤ 0.05; ^∗∗^*p* ≤ 0.01; ^∗∗∗^*p* ≤ 0.001).

Emodin promoted an increase in TBARS levels when compared with the control (DMSO 0.5%, *v*/*v*) in all cell lines, but in C33A and HSC-3, only the highest emodin concentration promoted a significant increase. Only the highest concentration of physcion caused a significant increase in TBARS levels in SiHa, C33A, and HaCaT, and no changes were observed in HSC-3. In SiHa, C33A, and HaCaT, EERs promoted an increase in TBARS, whereas in HSC-3, a lower concentration of EERs promoted a decrease in TBARS (Figure 6).

The redox status of transformed cells is significantly influenced by the levels of RONS such as O_2_^•**−**^, H_2_O_2_, ^•^NO, and HOCl/OCl^−^, and by the expression of enzymes such as NOX-1, SOD, and catalase in the cell membrane, promoting proliferation stimuli and inhibiting the deleterious effects of these reactive species. It is known that ^•^NO can induce cell death at some levels, as can the product of its reaction with O_2_^•**−**^, ONOO^−^. In addition, ^•^NO is able to inactivate catalase, leading to changes in the H_2_O_2_ levels; catalase can be reactivated by O_2_^•**−**^, releasing ^•^NO and the active enzyme, thus inducing cell death via HOCl/OCl^−^ and ^•^NO/ONOO^−^ [44, 45, 61]. We found that upon treating the cells with emodin and physcion, these compounds can interact with extra- and intracellular RONS (mainly O_2_^•**−**^ and ^•^NO), altering H_2_O_2_ and ^•^NO concentrations, thus unbalancing the performance of these enzymes. In addition to the effect on cell redox homeostasis, the formation of the respective semiquinones can also cause lipoperoxidation, corroborated by an increase in TBARS and heightened DNA damage.

Su et al. [50] observed a decrease in the cytotoxic effects of emodin after the pretreatment of A549 cells with the antioxidants ascorbic acid and N-acetyl cysteine. In addition, an increase in the concentrations of RONS promoted by the glycosylated form of physcion in HepG2 cells induced the activation of AMP-activated protein kinase (AMPK), resulting in the downregulation of DNA methyltransferase 1 (DNMT1), which is mediated by the transcription factor Sp1. DNMT1 is responsible for DNA methylation; its elevated expression has been reported to cause silencing of some tumor suppressor genes in various types of carcinomas [56].

### 3.5. Emodin, Physcion, and Chrysophanol Promoted Irreparable Damage to the DNA of Cervical Cancer Cells

The anthraquinones chrysophanol, emodin, and physcion were mutagenic only in SiHa and C33A cells after 24 h of treatment at all tested concentrations, except for chrysophanol, which was mutagenic only at a lower concentration. In HSC-3 and HaCaT cells, only the treatment with hydrogen peroxide (positive control) showed an increase in chromosomal aberrations. These results are shown in Table 3. HaCaT is a nontumoral immortalized cell line with p53 mutations that generate an unlimited proliferation phenotype [62]. On the other hand, HSC-3, SiHa, and C33A are tumoral lines, with high rates of genomic instability, intense proliferative characteristics, and high invasiveness capacities [5, 7, 8].

**Figure 7:**
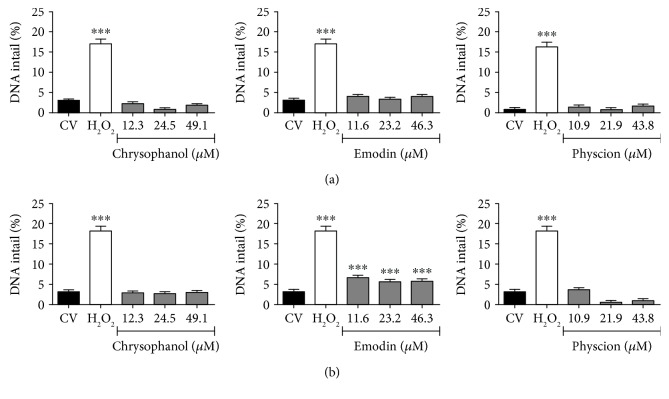
Comet assay for (a) C33A and (b) SiHa. Evaluation of genotoxicity of chrysophanol, emodin, and physcion. H_2_O_2_ (0.1 mM) as positive control and CV: vehicle control (1% DMSO). (^∗∗∗^ = statistically significant difference for *p* ≤ 0.001, analyzed by Kruskal-Wallis and Dunn's post-test).

The comet assay (Figure 7) was carried out in SiHa and C33A to verify the induction of reversible damage (DNA strand breaks). Only emodin showed significant genotoxicity in SiHa, but not in C33A cells. Taken together, data from the micronuclei and comet assays suggested that chrysophanol and physcion caused irreversible mutagenic damage by promoting errors during cell division (e.g., increase in micronuclei formation). On the other hand, emodin caused reversible breaks in DNA strands in the SiHa cell line, while also promoting chromosomal segregation errors (e.g., increase in micronuclei formation). Therefore, the damage promoted by emodin in SiHa cell DNA is more intense than that induced by chrysophanol and physcion. Furthermore, under conditions of oxidative stress, certain biomolecules are relevant targets of RONS, including DNA. The enzyme poly (ADP-ribose) polymerase 1 (PARP-1) binds to damaged DNA to catalyze protein ADP-ribosylation using NAD^+^ to initiate DNA repair. When the damage is limited, the DNA can be repaired and NAD^+^ levels are regenerated using ATP; however, if the DNA damage is not repaired, the cells die by apoptosis in a caspase-dependent manner. On the other hand, when damage is extensive, the sustained activation of PARP-1 depletes NAD^+^ and ATP stores; the cells are unable to activate the caspase pathway, which are ATP-dependent, and necrosis occurs [63]. Thus, the excessive activation of PARP-1 to repair the extensive DNA damage (verified in the micronuclei and comet assay), induced by the oxidative stress provoked by the treatment with emodin and physcion, may be related to caspase-independent cell death. Also, activation of PARP-1 increases the AMP/ATP ratio, activating the AMP-activated protein kinase (AMPK), which in turns inhibits mTORC1, leading to inhibition of anabolic processes and stimulation of catabolic events, which could also be contributing to cell death responses [64].

### 3.6. Emodin, Physcion, and EERs Changed the Levels of BAX and BCL-2 in SiHa Cells and the Levels of Caspase-3 in SiHa, C33A, HSC-3, and HaCaT Cells

**Figure 8:**
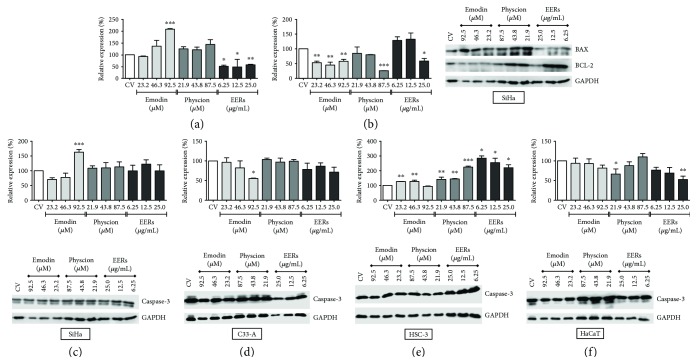
Levels of BAX, BCL-2, and caspases-3. (a) BAX, (b) BCL-2, (c) caspases-3 for SiHa, (d) caspases-3 for C33A, (e) caspases-3 for HSC-3, and (f) caspases-3 for HaCaT. Results are expressed as mean of electrophoretic bands densitometry, corrected for the control of cells treated with DMSO 0.5% (*v*/*v*) (CV), of two independent experiments ± standard error (*M* ± SE), analyzed by one-way ANOVA with Newman–Keuls post-test (statistically significant difference compared to CV: ^∗^*p* ≤ 0.05; ^∗∗^*p* ≤ 0.01; ^∗∗∗^*p* ≤ 0.001).

The levels of BAX and BCL-2 were studied only in the SiHa cell line, and the levels of caspase-3 were evaluated in all studied cells. In SiHa, the levels of BCL-2 (antiapoptotic protein) were decreased after treatment with emodin across all tested concentrations and after treatment with EERs at only the highest concentration, corroborating the cell death experiments where apoptosis and necrosis were observed. The levels of BAX (proapoptotic protein) were increased after treatment with 92.5 *μ*M emodin, whereas treatment with EERs caused a decrease in BAX levels (Figure 8). Furthermore, the decrease in BAX levels observed upon treatment with EERs may be related to autophagy-induced cell death, since the decrease in apoptotic modulators such as BAX has been associated with autophagic cell death. Known as type II cell death, it occurs via activation of cathepsins, without caspase activation [49]. Caspase-3 levels increased in the HSC-3 cells treated with emodin, physcion, and EERs. In SiHa, only emodin at its highest concentration increased the levels of caspase-3. Interestingly, emodin decreased the levels of caspase-3 in both C33A and HaCaT cells (Figure 8).

### 3.7. Emodin, Physcion, and EERs Inhibited AKT

**Figure 9:**
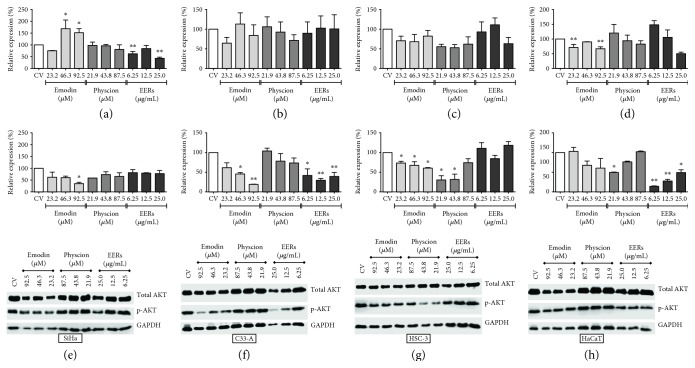
Total protein and phosphorylation (Ser-473 residue) levels of AKT. (a) Total AKT and (b) p-Ser-473-AKT for SiHa; (c) total AKT and (d) p-Ser-473-AKT for C33A; (e) total AKT and (f) p-Ser-473-AKT for HSC-3 (g); total AKT and (h) p-Ser-473-AKT for HaCaT. Results are expressed as mean of electrophoretic bands densitometry, corrected for the control of cells treated with DMSO 0.5% (*v*/*v*) (CV), of two independent experiments ± standard error (*M* ± SE), analyzed by one-way ANOVA with Newman–Keuls post-test (statistically significant difference compared to CV: ^∗^*p* ≤ 0.05; ^∗∗^*p* ≤ 0.01).

Emodin reduced the AKT phosphorylation levels of the serine-473 residue with at least one of the three tested concentrations in all tumor cell lines, most effectively in HSC-3, followed by C33A and SiHa, without promoting significant changes in the normal cell line (HaCaT) (Figure 9). Corroborating our findings, studies demonstrated that emodin promoted a decrease in the phosphorylation of AKT in A549 [50], SMMC-7721 [51], and HepG2 cells [52].

Physcion did not promote significant changes in AKT phosphorylation levels in cervical tumor cells (SiHa and C33A) but significantly reduced AKT phosphorylation in HSC-3 cells at the two lowest concentrations and in HaCaT cells at the lowest concentration (Figure 9). EERs reduced AKT phosphorylation in C33A and HaCaT cells at all tested concentrations; however, it did not promote significant changes in AKT phosphorylation in HSC-3 and SiHa cells (Figure 9).

The PI3K/AKT pathway is closely associated with proliferative signals triggered by mTOR (mammalian target of rapamycin). mTOR acts via two protein complexes: mTORC1, which is activated by AKT, and mTORC2, which in turn activates AKT via phosphorylation at the Ser-473 residue [65]. HSC-3 cells have mutations in PIK3CA that cause increases in AKT phosphorylation, while SiHa cells exhibit high expression levels of PIK3CA and, consequently, increased PI3K activity. The C33A cell line has a mutation in the catalytic subunit of PI3K which results in increased P13K activity and, consequently, strong AKT activation. C33A cells also have a mutation in PTEN, which introduces a premature stop codon in the gene, silencing its expression and thereby culminating in a further increase in the P13K and AKT activity [7]. Interestingly, emodin and EERs were able to reduce AKT activation in C33A cells, that is, in a cell line that is characterized by superstimulation of this pathway. Moreover, the imbalance in the PI3K/AKT pathway is associated with cell death in different tumor cells, thereby emerging as a potential target for cancer treatment [66–69].

## 4. Conclusion

**Figure 10:**
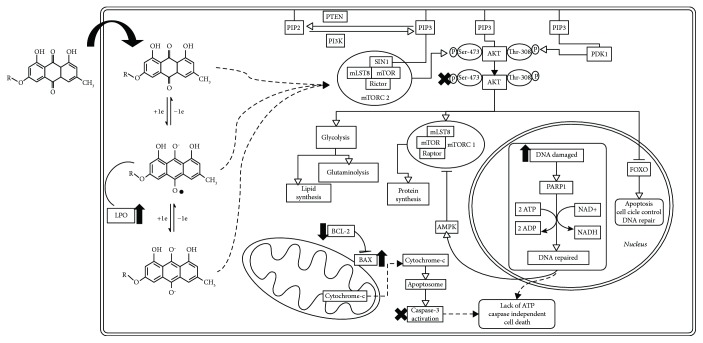
Hypothesis of the mechanisms causing cancer cell death induced by emodin and physcion. The intracellular oxidative environment stimulates the conversion of the anthraquinones (emodin, R=H, and physcion, R=OCH_3_) into their semiquinone forms, which may be causing both lipoperoxidation (LPO) and DNA damage and this last activating repair enzymes (e.g., PARP-1) and consequently increasing the ATP consumption. Anthraquinones could also be promoting an inhibition of metabolic processes important to cell survival and proliferation, via inhibition of AKT; this causes activation of FOXO factors, stimulation of cell cycle control, DNA repair, and activation of cell death pathways. The increased ATP consumption could also lead to AMPK activation, which inhibits anabolic pathways and activates catabolic processes. Taken together, the oxidative stress generated by the anthraquinones and the inhibition of AKT could both lead to loss of the metabolic profile favoring tumor survival and an imbalance in BAX and BCL-2 levels, resulting in induction of cell death via apoptosis and necrosis, independent of caspase-3 activation.

In summary, the anthraquinones emodin and physcion and the crude extracts and fractions of *Rhamnus sphaerosperma* var. *pubescens* induced cell death in both cervical and oral squamous cancer cells, likely by inducing multiple cellular events including increased oxidative stress, DNA damage, and inhibition of AKT (Figure 10). Taken together, the present findings highlight the promising anticancer activity of anthraquinones from *Rhamnus sphaerosperma*, supporting the need for ongoing investigations, mainly with emodin and physcion, contributing to the search of new therapeutic opportunities for cervical and oral squamous carcinomas.

## Figures and Tables

**Table 1:** Chemical shift values and coupling constants of ^1^H NMR spectra for chrysophanol, emodin, and physcion.

Position	Chemical shift (ppm) ^1^H		
	Chrysophanol	Emodin	Physcion
1 (C)–OH	12.09	12.21 (*s*)	12.10 (*s*)
2 (CH)	7.09 (*m*)	7.08 (*dq*; *J* = 1.6 Hz e 0.8 Hz)	7.07 (*m*)
3 (methyl)	2.46 (*s*)	2.45 (*dd*; *J* = 0.8 Hz e 0.5 Hz)	2.45 (*s*)
4 (CH)	7.65 (*m*)	7.60 (*dq*; *J* = 1.6 Hz e 0.5 Hz)	7.62 (*m*)
5 (CH)	7.81 (*d*; *J* = 7.8 Hz)	7.23 (*d*; *J* = 2.41 Hz)	7.35 (*d*; *J* = 2.61 Hz)
6 (CH)	7.68 (*dd*; *J* = 8.5 Hz e 7.8 Hz)	—	—
6 (methoxyl)	—	—	3.93 (*s*)
7 (CH)	7.28 (*d*; *J* = 8.5 Hz)	6.63 (*d*; *J* = 2.4 Hz)	6.67 (*d*; *J* = 2.60 Hz)
8 (C)–OH	12.11 (*s*)	12.31 (*s*)	12.29 (*s*)

*J* = coupling constant; *d* = doublet; *dd* = double doublet; *dq* = double quartet; *s* = singlet; *t* = triplet; *m* = multiplet.

**Table 2:** Capture capacity for O_2_^•−^, HOCl/OCl^−^, and ^•^NO.

	EC_50_ (*μ*g/mL)		
	O_2_^•−^	HOCl/OCl^−^	^•^NO
Curcumin	59.45 ± 5.85 *b*	0.5336 ± 0.081 *b*	15.69 ± 1.03 *b*
Gallic acid	18.81 ± 0.46 *c*	0.2843 ± 0.02 *b*	604.5 ± 52.52 *a*
Quercetin	13.48 ± 0.23 *c*	0.2594 ± 0.04 *b*	4.411 ± 1.69 *c*
Trolox	436.6 ± 9.39 *a*	4.578 ± 0.14 *a*	—
EERs	100.5 ± 0.68 *d*	3.195 ± 0.09 *c*	95.43 ± 8.73 *d*
HFRs	409.5 ± 26.36 *a*	4.99 ± 0.15 *a*	152.5 ± 4.11 *e*
CFRs	348.7 ± 12.02 *e*	3.028 ± 0.25 *c*	148.2 ± 10.34 *e*
EAFRs	144.7 ± 10.67 *d*	2.323 ± 0.12 *d*	49.7 ± 13.79 *f*

	EC_50_ (*μ*M)		
	O_2_^•**−**^	HOCl/OCl^−^	^•^NO
Curcumin	112.9 ± 11.14 *b*	1.014 ± 0.15 *b*	30.62 ± 2.01 *b*
Gallic acid	110.6 ± 2.72 *b*	1.672 ± 0.10 *b*	3556 ± 309 *a*
Quercetin	44.61 ± 0.75 *c*	0.8583 ± 0.14 *b*	14.59 ± 5.58 *b*
Trolox	1813 ± 52.81 *a*	17.82 ± 0.55 *a*	—
Emodin	140.8 ± 1.44 *d*	26.56 ± 0.74 *c*	52.03 ± 4.28 *c*
Physcion	—	33.1 ± 4.06 *c*	377 ± 16.71 *d*
Chrysophanol	—	23.56 ± 1.12 *c*	243 ± 18.3 *e*

Results are expressed as mean of the EC_50_ ± standard error (*M* ± SE), analyzed by one-way ANOVA with Tukey's post-test (different letters represent statistical difference) (− = no activity).

**Table 3:** Micronuclei assay.

	Micronuclei frequency (%)			
	SiHa	C33A	HSC-3	HaCaT
DMSO (1%, *v*/*v*)	0.939 ± 0.09	0.617 ± 0.03	4.16 ± 0.54	0.177 ± 0.02
H_2_O_2_ (0.1 mM)	1.916 ± 0.32^∗^	1.190 ± 0.16^∗∗^	9.33 ± 0.63^∗^	1.198 ± 0.27^∗∗∗^
*Chrysophanol*				
3.125 *μ*g/mL (12.3 *μ*M)	3.894 ± 0.33^∗∗∗^	0.757 ± 0.20	3.913 ± 0.69	0.228 ± 0.18
6.25 *μ*g/mL (24.5 *μ*M)	2.832 ± 0.21^∗∗∗^	1.129 ± 0.05^∗∗^	2.655 ± 0.063	0.066 ± 0.02
12.5 *μ*g/mL (49.1 *μ*M)	2.431 ± 0.2^∗∗^	1.403 ± 0.07^∗∗∗^	2.706 ± 0.9	0.151 ± 0.09
*Emodin*				
3.125 *μ*g/mL (11.6 *μ*M)	2.120 ± 0.13^∗^	0.932 ± 0.08^∗^	4.720 ± 0.82	0.181 ± 0.06
6.25 *μ*g/mL (23.2 *μ*M)	2.428 ± 0.36^∗∗^	1.318 ± 0.13^∗∗∗^	4.246 ± 1.23	0.296 ± 0.16
12.5 *μ*g/mL (46.3 *μ*M)	3.528 ± 0.19^∗∗∗^	1.081 ± 0.04^∗∗^	2.789 ± 1.63	0.102 ± 0.018
*Physcion*				
3.125 *μ*g/mL (10.9 *μ*M)	1.699 ± 0.26^∗^	1.298 ± 0.06^∗∗^	5.723 ± 0.96	0.478 ± 0.21
6.25 *μ*g/mL (21.9 *μ*M)	1.990 ± 0.27^∗^	1.246 ± 0.12^∗∗^	4.794 ± 1.02	0.421 ± 0.091
12.5 *μ*g/mL (43.8 *μ*M)	1.896 ± 0.17^∗^	1.143 ± 0.15^∗∗^	4.915 ± 0.76	0.239 ± 0.19

Hydrogen peroxide (0.1 mM) as positive control and DMSO (1%, *v*/*v*) as vehicle control. Mean ± standard error of the number of micronuclei found in three independent experiments on cells. All cells were analyzed by the IN Cell Analyzer 2000 (GE Healthcare) software. (^∗^*p* ≤ 0.05; ^∗∗^*p* ≤ 0.01; ^∗∗∗^*p* ≤ 0.001 compared to vehicle control analyzed by ANOVA and Newman–Keuls post-test).

## Data Availability

The authors confirm that the data supporting the findings of this study are available within the article.

## Abbreviations

EERs: Ethanolic crude extract from stems of *Rhamnus sphaerosperma* var. *pubescens*
HFRs: Hexanic fraction
CFRs: Chloroform fraction
EAFRs: Ethyl acetate fraction
PI3K: Phosphatidylinositol-3-kinase
AKT: Protein kinase B
PTEN: Phosphatase and tensin homolog
PDK-1: Pyruvate dehydrogenase kinase 1
mTORC2: Mammalian target of rapamycin complex 2
RONS: Reactive oxygen and nitrogen species
SOD: Superoxide dismutase
NOX-1: NADPH oxidase 1
DUOX-POD: Dual oxidase with peroxidase domain
O_2_^•**−**:^: Superoxide anion
H_2_O_2_: Hydrogen peroxide
^•^NO: Nitric oxide
HOCl/OCl^−^: Hypochlorous acid/hypochlorite anion
TBARS: Thiobarbituric acid reactive species.
